# Calcium Hydroxylapatite‐Carboxymethylcellulose as a Treatment for Forearm Dermatoporosis: A Prospective, Delayed‐Start, Contralateral Case Series

**DOI:** 10.1111/jocd.70859

**Published:** 2026-04-12

**Authors:** Ada Regina Trindade de Almeida, Maria Cortez de Sousa Marins Barbosa, Alexandre Michalany, Alec D. McCarthy

**Affiliations:** ^1^ Clinica de Dermatologia Hospital Do Servidor Municipal de São Paulo São Paulo Brazil; ^2^ Merz Aesthetics Raleigh North Carolina USA

## Abstract

**Background:**

Dermatoporosis is a chronic cutaneous fragility syndrome marked by dermal thinning, collagen loss, and recurrent purpuric lesions. Effective treatments remain limited. Calcium hydroxylapatite–carboxymethylcellulose (CaHA–CMC) is a regenerative biostimulator with demonstrated skin extracellular matrix–restorative effects, but its usefulness in forearm dermatoporosis has not been established.

**Objectives:**

To evaluate the efficacy, durability, and safety of hyperdiluted CaHA–CMC for reducing lesion burden, increasing dermal thickness, and enhancing collagen density in dermatoporotic forearms.

**Methods:**

In this prospective, delayed‐start, contralateral case series, six patients with bilateral dermatoporosis received a single treatment of 1:2 CaHA–CMC. The more clinically affected arm was treated at Day 0, and the contralateral arm served as a 45‐day untreated control before receiving identical treatment. Lesion coverage was quantified from standardized photographs using an ImageJ‐based workflow. Dermal thickness was measured by high‐frequency ultrasound, and collagen content was assessed from Masson's Trichrome–stained punch biopsies. Early effects (Day 0–45) were evaluated using paired testing and a subject‐level difference‐in‐differences (DiD) estimator. Durability was assessed by aligning post‐treatment follow‐ups by time‐since‐treatment (TST), focusing on TST45 and an ~1‐year window (320–365 days).

**Results:**

From Day 0 to Day 45, lesion coverage decreased in treated forearms from 5.60% ± 4.47% to 1.92% ± 2.07% (*p* = 0.041), while changes in untreated contralateral forearms were not significant. In pooled participant‐level analyses aligned by post‐treatment timing, lesion coverage decreased from 4.27% ± 1.88% at baseline to 1.16% ± 1.10% at TST45 (*p* = 0.0313) and remained reduced at the ~1‐year window (1.14% ± 0.68%, *p* = 0.0313), corresponding to a 73.4% reduction at ~1 year. Collagen area increased by 69.7% at 45 days post‐treatment (*p* = 0.0009), and dermal thickness increased by 36.05% (*p* = 0.0007). Treatment was well tolerated; one transient nodule resolved with massage.

**Conclusions:**

A single session of hyperdiluted CaHA–CMC produced rapid and durable clinical, histologic, and ultrasonographic improvement in forearm dermatoporosis. Findings warrant further evaluation in larger controlled trials.

## 1 Introduction

Previously described as Bateman's senile purpura, the term dermatoporosis was first introduced in 2007 by Kaya and Saurat to describe a skin fragility syndrome associated with aging [1] Fibroblast senescence contributes to the loss of the dermal extracellular matrix and its main structural components including collagen and elastin [2, 3] This structural degradation reduces the skin's mechanical integrity, which further exacerbates collagen loss and accelerates signs of aging [4]. Dermatoporosis manifests in various forms, including atrophic skin, purpuric lesions, and star‐shaped scars, predominantly in photo‐exposed areas such as the extensor surface of the forearms, dorsum of the hands, and thighs [5].

Two types of dermatoporosis are classified by their etiology: primary and secondary. The primary and most common type results from skin aging, genetic factors, and chronic sun exposure [1]. The secondary type is generally linked to prolonged use of corticosteroids, as well as conditions like chronic kidney failure, chronic obstructive pulmonary disease, and the use of epidermal growth factor receptor inhibitors or anticoagulants. While clinical manifestations of both forms are similar, ultrasonography reveals greater atrophy in the secondary type, which often presents more severely and earlier in life [6]. The onset of dermatoporosis typically occurs between ages 40 and 60, fully progressing by ages 70–90, and is more prevalent in women, with aging being the most significant risk factor [7].

Diagnosis is primarily clinical (i.e., dark purple to reddish‐brown patches purpuric in appearance), but histological analysis reveals a thinned epidermis and a dermis characterized by decreased collagen fibers sometimes replaced by abnormal elastic fibers [1]. The vascular structures remain intact, but the loss of peripheral support causes red blood cell extravasation and hemosiderin deposition. In ultrasonographic studies, a subepidermal low echogenic band (SLEB) serves as a marker of photo‐aging and dermatoporosis [8]. The thickness of atrophic skin affected by dermatoporosis is reduced to 0.7–0.8 mm, compared to normal skin thickness generally exceeding 1 mm [1, 6].

Dermatoporosis can be classified into four clinical stages, with Stage I further subdivided based on lesion count [1]. Stage I is defined by the presence of senile purpura and/or pseudoscars, without skin lacerations. It is subdivided into: Stage IA, characterized by fewer than 10 pseudoscars and fewer than 10 purpuric lesions; Stage IB, with fewer than 10 pseudoscars and more than 10 purpuric lesions; Stage IC, with more than 10 pseudoscars and fewer than 10 purpuric lesions; and Stage ID, where both pseudoscars and purpura exceed 10 lesions. Stage II is marked by the appearance of fewer than 10 superficial skin lacerations, indicating the onset of functional skin fragility. In Stage III, patients exhibit more than 10 lacerations, reflecting more widespread trauma‐induced injury. Stage IV is defined by the presence of any dissecting hematoma, representing a severe complication due to subcutaneous bleeding and requiring prompt medical attention. Effective treatment options are limited, with photoprotection serving as one of the only known preventive measures. Other proposed interventional therapies include intense pulsed light, topical retinaldehyde, hyaluronate fragments, epidermal growth factor creams, bioflavonoids, topical dehydroepiandrosterone (DHEA), and vitamin C supplementation, and the use of injectable biostimulators [7, 9, 10].

Calcium hydroxylapatite‐carboxymethylcellulose (CaHA‐CMC; Radiesse, Merz Aesthetics Inc) is both a filler and regenerative biostimulator [11]. When undiluted, CaHA‐CMC directly volumizes soft tissue. When diluted (1:1) or hyperdiluted (1:> 1), the direct volumizing capacity is almost entirely eliminated and the primary corrective effect results from dermal extracellular matrix (ECM) regeneration [12]. Histological and immunohistochemical studies have demonstrated increased types I and III collagen, elastin, and proteoglycans following injections of diluted and hyperdiluted CaHA‐CMC [13, 14]. Positive improvements in the epidermis, including decreased keratinization, improved hydration, and decreased dyspigmentation, have also been previously reported [15].

Importantly, evidence from anatomically comparable upper‐limb sites supports translation of CaHA‐CMC biostimulation to photoexposed, mechanically fragile skin. In a prospective upper arm study, a single session of hyperdiluted (1:4) CaHA‐CMC produced visible photographic improvement and a measured increase in hydration by corneometry at 60 days [16]. In a split‐side arm study comparing CaHA and PLLA, superficial subcutaneous/subdermal injections of CaHA‐CMC were associated with clinical improvement and histologic evidence of collagen and elastic fiber neoformation in the subcutaneous tissue adjacent to biostimulator particles [17].

Building on these skin‐quality data, in 2018 Souza and Serra first proposed CaHA‐CMC as a therapeutic approach for actinic purpura lesions—often considered an early manifestation within the dermatoporosis spectrum—reporting improvement in clinical severity and structural support [18]. More recently, Seo et al. reported improvement in senile purpura on the dorsum of the hand following subdermal injection of 1:1 diluted CaHA‐CMC using a proximal‐to‐distal fanning technique, accompanied by histologic increases in collagen and elastic fibers and increased dermal YAP expression, suggesting a mechanotransduction‐mediated pathway relevant to dermal reinforcement in purpura‐prone skin [19]. Consistent with these upper‐limb observations, ultrasound‐based studies in other photoexposed regions (e.g., the neck) have demonstrated increased dermal thickness following hyperdiluted CaHA‐CMC [20]. Collectively, the existing literature supports biological plausibility for CaHA‐CMC as a regenerative intervention for forearm dermatoporosis; however, available studies have largely emphasized skin quality, laxity, and hydration outcomes rather than quantifying dermatoporosis lesion burden and durability, leaving key evidence gaps.

Therefore, the primary objective of this study was to evaluate the efficacy, duration, and safety of diluted CaHA‐CMC injections for treating dermatoporosis in the forearms. Secondary objectives included reducing the area of purpuric lesions, decreasing skin atrophy as measured by high‐frequency ultrasound, and improving dermal collagen content through histological analysis of skin biopsies.

## 2 Materials and Methods

This open‐label, prospective case series included patients from the dermatology clinic at Hospital do Servidor Público Municipal de São Paulo, Brazil (CAAE: 66468822.8.0000.5442). Inclusion criteria required a clinical diagnosis of dermatoporosis in the forearms, with no prior treatments for at least 30 days. Exclusion criteria included anticoagulant or antiplatelet use, systemic or topical corticosteroids or immunosuppressants, and allergies to lidocaine, chlorhexidine, or CaHA‐CMC. A full list of exclusion criteria is found in Table S1. Six patients were enrolled, and written informed consent was obtained.

Each participant contributed two study arms: the more clinically severe arm (immediate‐treatment arm) and the less severe forearm (delayed‐start arm). The immediate‐treatment arm received CaHA‐CMC at Day 0. The delayed‐start arm served as an untreated contralateral comparator through Day 45 and then received identical treatment at Day 45. Clinical assessments and standardized photography were obtained at Days 0, 45, 90, and 365 for both forearms. This delayed‐start contralateral design provided a 45‐day natural‐history control period and permitted all patients to ultimately receive treatment, consistent with ethical delayed‐start paradigms.

### 2.1 Technical Procedures

At Day 0, baseline assessments included lesion counts, skin quality evaluations using the pinching test by two evaluators, photographic documentation, and marking reference points for evaluations. High‐frequency ultrasound was performed on both forearms at baseline (Day 0) taken at Point 2. A 4‐mm punch biopsy was obtained at Point 1 from the immediate‐treatment forearm only, prior to treatment for baseline histology. Histological stains included Masson's Trichrome (collagen) and hematoxylin and eosin (H&E) stains.

**FIGURE 1:**
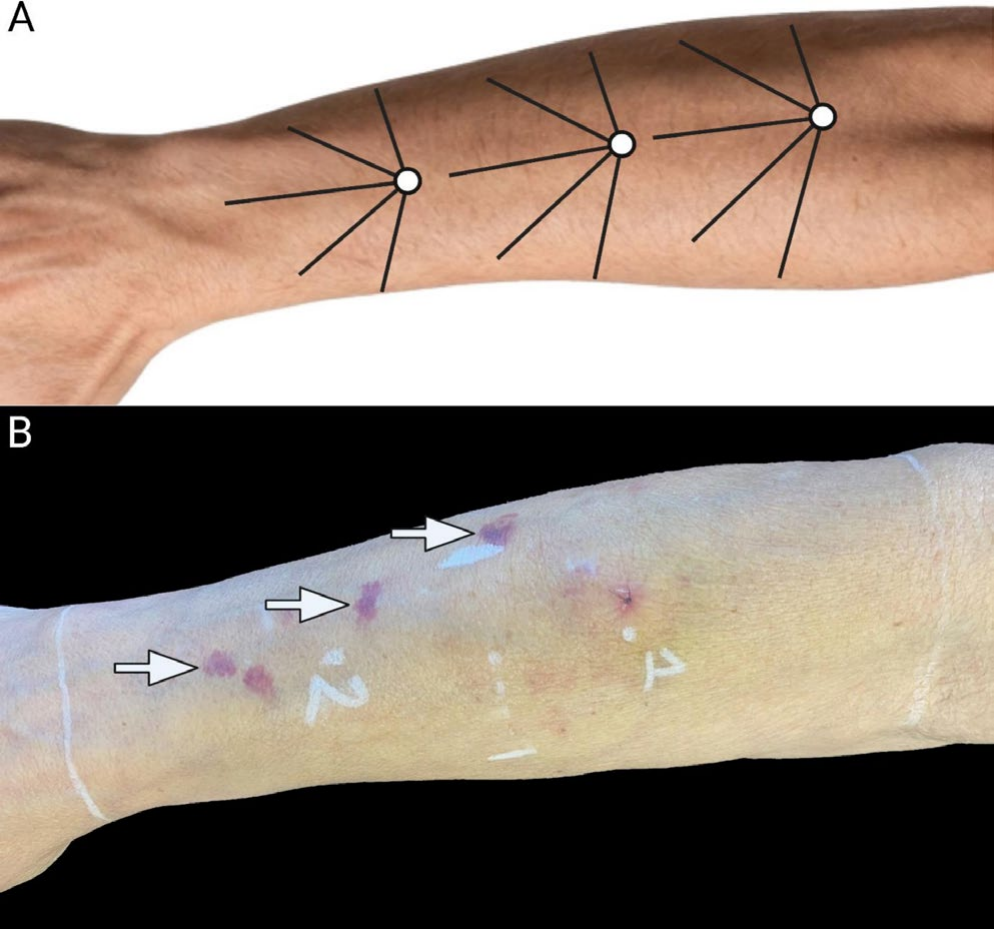
Standardized forearm injection mapping and representative dermatoporotic forearm showing characteristic lesions and injection landmarks used in this study. (A) Schematic of the injection pattern used in this study. The forearm was divided into three segments, and hyperdilute CaHA‐CMC was delivered into the subdermal plane using a fanning approach with retrograde linear threading. (B) Representative clinical photograph of a patient with Stage 1C dermatoporosis demonstrating multiple purpuric lesions (white arrows), atrophic dorsal forearm skin, and study markings. White lines demarcate the proximal and distal boundaries of the treatment area. Point 1 indicates the biopsy site and Point 2 indicates the ultrasound measurement site.

At Day 0, the immediate‐treatment forearms received two 1.5 mL syringes of CaHA‐CMC (total product volume = 3.0 mL). A single treatment session was performed as a proof‐of‐concept regimen to evaluate objective clinical and structural endpoints. A 1:2 dilution (product: diluent) was defined as one‐part CaHA‐CMC combined with two parts diluent; accordingly, 3.0 mL of diluent was added to each 1.5 mL syringe. Specifically, CaHA‐CMC was diluted with 2.5 mL of 0.9% bacteriostatic normal saline and 0.5 mL of 2% lidocaine for a final injectate volume of 4.5 mL per syringe (9.0 mL total injectate per forearm). The forearm was divided into three segments for injection. After topical anesthesia, injections were performed with a 25‐gauge cannula in the subdermal plane (targeting the deep dermal‐superficial subcutaneous interface, immediately deep to the dermis) using a distal‐to‐proximal fanning technique with retrograde linear threading (Figure 1A). Product distribution was standardized as three longitudinal series along the forearm (3.0 mL per series), with each series comprising five retrograde threads of 0.6 mL (15 total threads per forearm) (Figure 1B). No specific post‐treatment instructions were given to the patients.

At Day 45, the immediate‐treatment forearm underwent reassessment including photography and high‐frequency ultrasound, and post‐treatment biopsy was obtained from the immediate‐treatment forearm (TST 45). The delayed‐start forearm then received the same treatment protocol at Day 45.

High‐frequency ultrasound was repeated 45 days after treatment for each forearm (TST 45): at Day 45 for the immediate‐treatment forearm and at Day 90 for the delayed‐start forearm. Long‐term follow‐up assessments were performed at approximately 1 year after enrollment (Day 365). Because the delayed‐start forearm was treated 45 days later, its Day 365 visit corresponded to approximately 320 days post‐treatment; thus, 320‐ and 365‐day assessments were summarized within an ~1‐year durability window.

Post‐treatment biopsies were performed at Day 45 to interrogate early‐to‐mid stage dermal remodeling after CaHA‐CMC at which treatment‐related changes such as early collagen remodeling and increases in dermal thickness may be detectable, while maintaining the delayed‐start control period and minimizing attrition for primary objective analysis [13, 21]. This timepoint was not intended to capture peak neocollagenesis, which prior literature suggests may occur later [15, 21].

### 2.2 ImageJ Lesion Analysis

Computer‐assisted image analysis (CAIA) workflows using ImageJ have been widely applied in dermatology to quantify lesion area and color‐based skin findings from clinical photographs, supporting the feasibility of objective image‐derived endpoints [22, 23]. Notably, ImageJ‐based CAIA has been used to quantify lesion area and erythema‐related color changes in inflammatory dermatoses, supporting customization of this approach for quantifying dermatoporosis lesion burden [24]. Accordingly, we implemented a study‐specific ImageJ FIJI workflow with prespecified parameters to quantify dermatoporosis lesion burden, as detailed below:

Images of dermatoporotic lesions on the arm were analyzed using ImageJ FIJI (National Institutes of Health, Bethesda, MD). Initially, the images were loaded into ImageJ, and the Wand Tool was employed to select regions of interest (ROIs) with a tolerance threshold of 70 to delineate each patient's arms. ROIs were saved by right‐clicking on the selection and choosing Add to ROI Manager, followed by naming and saving each ROI for area correction. Background content was removed using the Edit>Clear Outside function.

**FIGURE 2:**
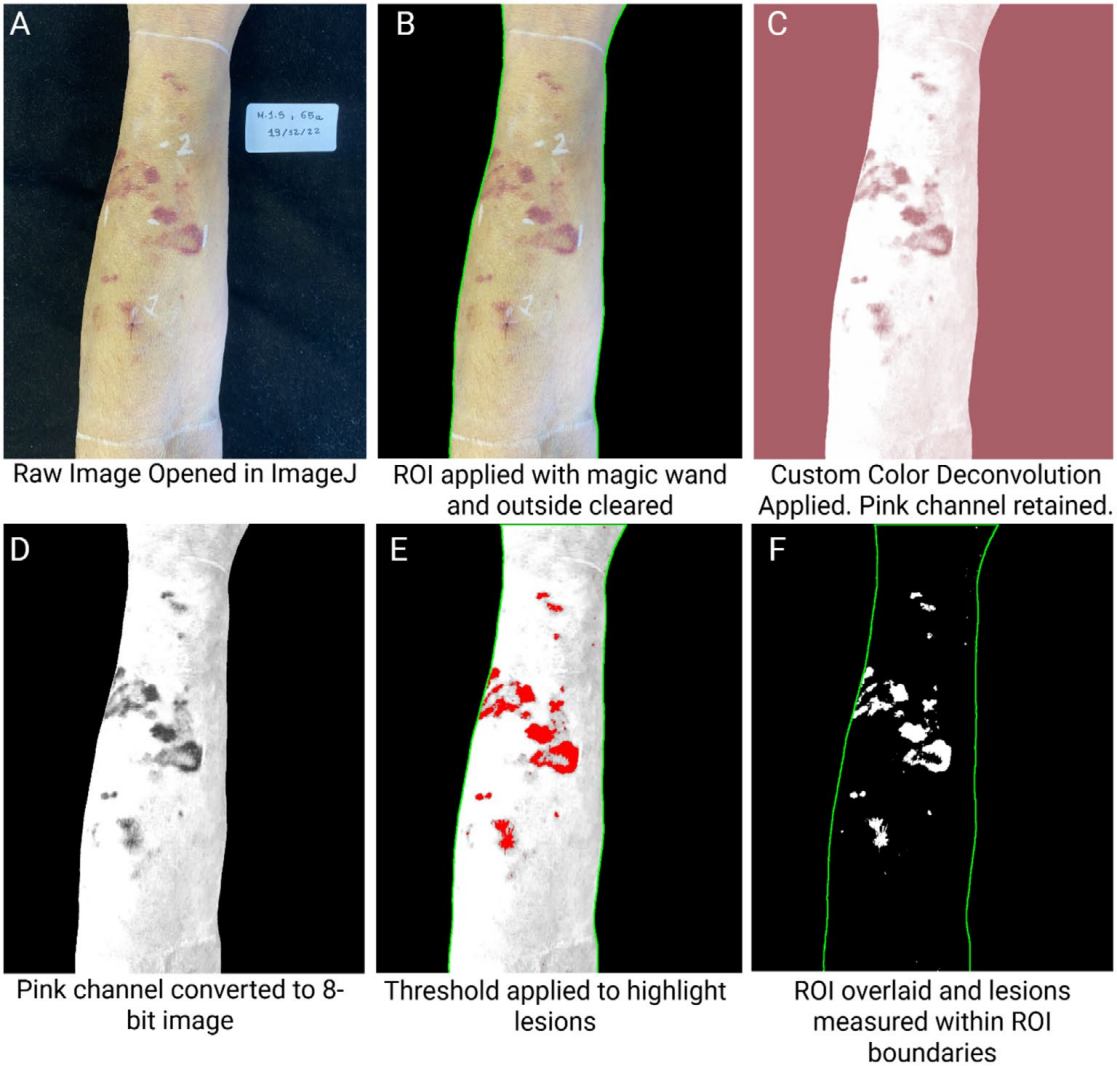
Workflow for lesion quantification using ImageJ‐based image processing. (A) Original clinical photograph of a dermatoporotic forearm with purpuric lesions. (B) Region of interest (ROI) defined using the Wand Tool in ImageJ to isolate the forearm and remove background content. (C) Application of Color Deconvolution to emphasize red and purple hues characteristic of dermatoporotic lesions. The pink channel was retained for optimal contrast. (D) Grayscale conversion of the pink channel to facilitate threshold‐based segmentation. (E) Lesion areas highlighted in red following threshold adjustment and binarization. (F) Final binary mask of isolated lesions used to calculate lesion relative to the ROI.

To reduce the influence of global lighting and color‐balance variation and to isolate lesion‐specific color components, Color Deconvolution was applied with a custom color deconvolution matrix tailored to emphasize the red and purple hues characteristic of the lesions. The matrix parameters were: [0.3589788, 0.6791887, 0.640185, 0.3685909, 0.4828154, 0.7943205, 0.7064397, 0.5340295, 0.4642794]. From the resulting deconvoluted channels, the pink channel was retained as it provided the optimal contrast for isolating the lesions. The retained pink channel was then converted to an 8‐bit grayscale image (Image>Type > 8‐bit) to facilitate threshold‐based segmentation. A uniform global threshold was applied (Adjust>Threshold) to further isolate the lesions, and the thresholded mask was finalized by selecting Apply. The final binary image was subjected to measurement using the Analyze>Measure function, with output parameters set to capture % Area of the lesions relative to the total ROI. This process provided quantitative data on lesion size and coverage percentage. Results were recorded and saved for further statistical analysis. A visual workflow is presented in Figure 2.

### 2.3 Histological Analysis

Two 4‐mm punch biopsies were obtained from each participant's immediate‐treatment forearm at a standardized reference location (Point 1, Figure 1B) to enable within‐subject comparisons over time: one at baseline (Day 0, prior to treatment) and one at Day 45 (TST 45). To minimize cannula‐tract artifact and biopsy‐induced remodeling at the sampling site, follow‐up biopsies were collected using a standardized approach from a non‐overlapping site adjacent to the pre‐marked reference Point 1 while avoiding cannula entry points and visible injection tracks; baseline and follow‐up biopsies were not obtained from the same puncture site. Histological processing and staining were performed using a standardized protocol, and slides were imaged under consistent microscope/camera settings at the same magnification across timepoints.

Histological images of the same magnification from each timepoint were analyzed using an ImageJ FIJI macro adapted from established protocols [25, 26]. Images stained with Masson's Trichrome (MT) for collagen were batch‐processed from defined file paths. Each image underwent color deconvolution with the preset MT channel, and the high‐contrast green channel was selected for thresholding to isolate stained fibers. Following application of a binary mask, integrated density and percent area were quantified to assess signal intensity and stained coverage. Quantitative analyses were performed using prespecified processing and thresholding parameters applied uniformly across specimens. Images were coded and analyzed blinded to forearm and timepoint.

### 2.4 Ultrastructural Analysis

For ultrasound‐measured dermal thickness, data are presented as mean ± standard deviation (SD). Measurements were obtained at baseline (Day 0) in both forearms and repeated 45 days post‐treatment for each forearm (immediate‐treatment forearm: Day 45; delayed‐start forearm: Day 90). Within‐forearm changes from baseline were assessed using paired two‐tailed *t*‐tests, with the Wilcoxon signed‐rank test used as a nonparametric sensitivity analysis when normality assumptions were not met.

### 2.5 Statistical Analysis

Statistical significance thresholds were defined as: ns = *p* > 0.05, *p < 0.05, **p < 0.01, ***p < 0.001, ****p < 0.0001. All analyses were performed using GraphPad Prism v10.2.3 (GraphPad LLC, San Diego, CA). Given the small sample size, nonparametric tests were prioritized for paired comparisons, and *p*‐values for longitudinal analyses were interpreted as exploratory.

### 2.6 Early Intra‐Arm (Delayed Start) Analysis

Early changes in lesion burden over the initial 45‐day observation period were compared between the immediate‐treatment and delayed‐start, within‐subject framework. For each participant, the change in lesion coverage (% area) from Day 0 to Day 45 was calculated for the immediate‐treatment forearm and for the contralateral delayed‐start forearm (untreated during this window). The primary early comparative estimator was subject‐level difference‐in‐differences (DiD) defined as:

$${\mathrm{DiD}}_{i}=\left({\text{Immediate}}_{i,\mathrm{Day}45}-{\text{Immediate}}_{i,\mathrm{Day}0}\right)-\left({\text{Delayed}}_{i,\mathrm{Day}45}-{\text{Delayed}}_{i,\mathrm{Day}0}\right)$$

The DiD distribution was tested against zero using a one‐sample Wilcoxon signed‐rank test (two‐tailed, exact). Within‐arm Day 0 versus Day 45 changes were assessed using paired two‐tailed *t*‐tests, with Wilcoxon signed‐rank tests used as nonparametric sensitivity analyses when appropriate.

### 2.7 Pooled Long‐Term (Time‐Since‐Treatment) Analysis

After Day 45, both arms had received treatment. To enable longitudinal evaluation, all observations were re‐indexed by time‐since‐treatment (TST), with baseline defined as Day 0 (pre‐treatment for both forearms) and follow‐up aligned to each forearm's treatment day. The immediate‐treatment arm contributed data at baseline, TST 45, and TST 365 days, and the delayed‐start arm contributed at baseline, TST 45, and TST 320 days. For pooling, the 320‐ and 365‐day measurements were analyzed jointly as a ~1‐year window. Longitudinal inference accounted for within‐subject clustering by using participant‐level summaries at each TST (and paired comparisons versus baseline); *p*‐values for pooled trajectories were interpreted as exploratory given the sample size and repeated testing.

An omnibus time effect across pooled aligned timepoints was evaluated using a mixed‐effects model with participant random intercept and time as a categorical fixed effect, with model comparison performed versus an intercept‐only model (likelihood‐ratio test).

One participant was missing the Day 45 follow‐up and was therefore excluded from the 0–45‐day intra‐arm analysis but retained for later TST assessments.

### 2.8 Arm‐Specific Time Course Analysis

Arm‐specific lesion trajectories were evaluated separately for the immediate‐treatment and delayed‐start forearms after re‐indexing observations by time‐since‐treatment (TST). For each forearm, lesion coverage (% area) was summarized at each available TST timepoint. Within‐arm changes were assessed relative to each arm's baseline using paired two‐tailed tests (*t*‐test; Wilcoxon signed‐rank as sensitivity). Between‐arm comparisons at matched TST timepoints (TST = 0, 45, and ~1 year) were performed using paired tests to account for within‐subject correlation between contralateral forearms. TST 90 was not available for the delayed‐start forearm (no Day 135 visit) and therefore was analyzed for the immediate‐treatment forearm only. These analyses were designed to evaluate whether response trajectories differed by arm after alignment to time‐since‐treatment.

Overall time effects within each forearm were additionally evaluated using mixed‐effects models fit separately for the immediate‐treatment and delayed‐start forearms, with subject random intercept and day as a categorical fixed effect, and model comparison performed versus an intercept‐only model (likelihood‐ratio test).

## 3 Results

Six patients were enrolled, with baseline characteristics including a history of dermatoporosis due to aging, weight loss, or prior steroid use occurring before study enrollment. The average age was 67.33 ± 1.751 years, and 5/6 subjects were female. At the stage of enrollment, 3/6 patients were diagnosed with Stage 1C while 2/6 were diagnosed with Stage 1B, and 1/6 was diagnosed with Stage 1D dermatoporosis.

### 3.1 Photographic Analysis

**FIGURE 3:**
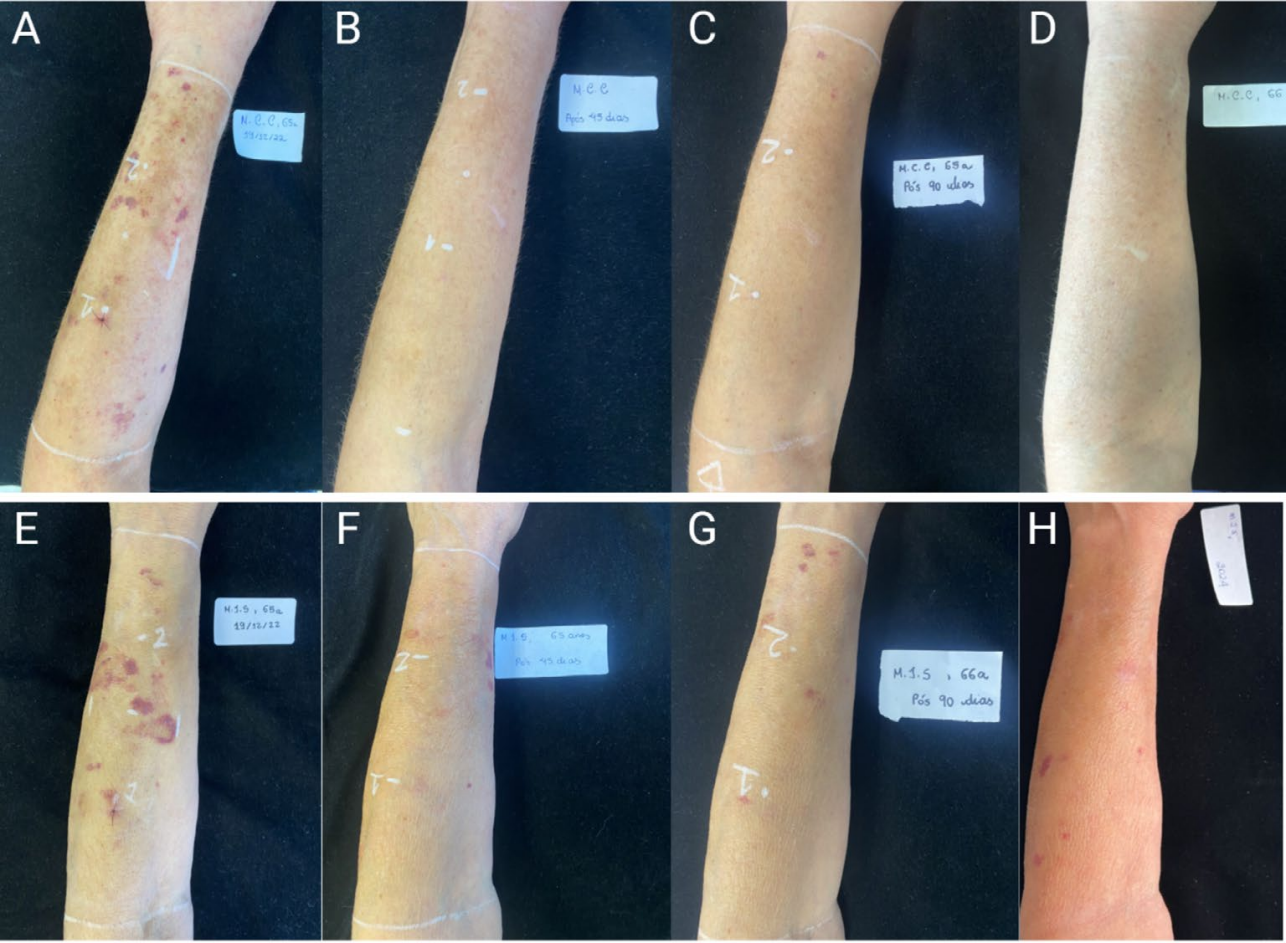
Clinical progression of two patients treated with hyperdiluted CaHA–CMC over 12 months. Representative serial photographs of two patients (top row: A–D; bottom row: E–H) at four timepoints following treatment. (A, E) Baseline, showing multiple purpuric and atrophic lesions characteristic of forearm dermatoporosis. (B, F) Forty‐five days post‐treatment, demonstrating a visible reduction in lesion number and erythema with early improvement in dermal tone. (C, G) Ninety days post‐treatment, showing continued fading of lesions and smoother, more uniform skin texture. (D, H) Approximately 1‐year post‐treatment, illustrating sustained improvement with near‐complete resolution of purpura and restoration of normal pigmentation and skin quality.

Clinical progression showed sustained improvements in patients marked by decreases in the size and intensity of lesions, resulting in an overall improvement of the visible signs of dermatoporosis (Figure 3). No patient showed an increase in lesion count or area throughout the time course of the study.

### 3.2 Lesion Burden Analysis

#### 3.2.1 Early Intra‐Arm (Delayed‐Start) Comparison

During the first 45 days, only the more severely affected forearms received CaHA‐CMC, while the contralateral, less‐severe arms served as untreated controls. Lesion coverage in the treated arms decreased from 5.60% ± 4.47% at baseline to 1.92% ± 2.07% at Day 45, compared with a reduction from 2.95% ± 1.67% to 1.80% ± 1.49% in the untreated arms. Reductions within the immediate‐treatment arm were statistically significant (*p* = 0.041), whereas reductions within the delayed‐treatment arm prior to receiving intervention were not (*p* = 0.289).

A subject‐level DiD analysis favored treatment, with a mean DiD of −2.89% (negative values indicating greater improvement in the treated forearm). However, the DiD did not differ significantly from zero (one‐sample Wilcoxon signed‐rank test, two‐tailed exact *p* = 0.1875), indicating that early between‐arm differences should be interpreted cautiously given sample size and variability. Although not statistically significant, the direction and magnitude of the DiD effect support the interpretation that early improvements were attributable to treatment rather than spontaneous fluctuation.

#### 3.2.2 Pooled Time‐Since‐Treatment (TST) Trajectory

**FIGURE 4:**
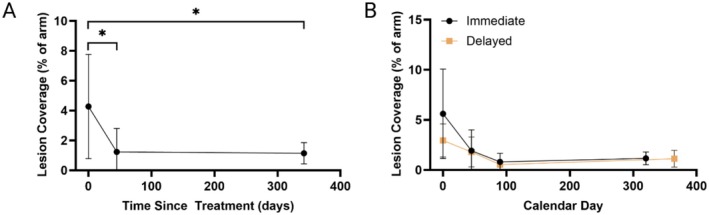
Quantitative reduction in dermatoporotic lesion coverage following treatment with hyperdiluted CaHA–CMC. (A) Pooled time‐since‐treatment and (B) single‐arm trajectory showing significant and durable improvement following CaHA‐CMC injection. *p < 0.05.

After Day 45, both arms had received treatment. For longitudinal assessment, all visits were re‐indexed relative to each arm's treatment day (TST 0, TST 45, and TST ~1 year). When data were pooled across all arms, mean lesion coverage decreased from 4.27% ± 1.88% at baseline (TST 0) to 1.16% ± 1.10% at TST 45 (*p* = 0.0313), and 1.14% ± 0.71% at approximately 1 year (pooled 320–365 days, *p* = 0.0313; Figure 4A). This corresponds to a 73.4% overall reduction in lesion coverage at TST ~1 year relative to baseline. Pairwise comparisons versus baseline demonstrated significant improvement by TST 45 and sustained benefit through TST ~1 year. These findings indicate an early response to treatment followed by long‐term stabilization of skin quality and lesion resolution.

Consistent with these pairwise comparisons, a mixed‐effects model with participant random intercept and TST as a categorical fixed effect showed a significant overall time effect across the pooled trajectory (likelihood‐ratio test vs. intercept‐only model, *p* = 0.0001), supporting a sustained reduction in lesion burden following treatment.

#### 3.2.3 Arm‐Specific Time Courses

Arm‐specific trajectories demonstrated durable reductions in dermatoporotic lesion burden following CaHA–CMC treatment (Figure 4B). In the immediate‐treatment forearm, lesion coverage declined from 5.60% ± 4.47% at baseline (Day 0) to 1.92% ± 2.07% at Day 45, with further reductions observed at Day 90 (0.81% ± 0.84%) and sustained improvement at Day 365 (1.15% ± 0.68%). Paired comparisons versus Day 0 showed directional improvement at Day 45 (*p* = 0.0625) and Day 90 (*p* = 0.0625), with a significant reduction maintained at Day 365 (*p* = 0.0313). Consistent with these findings, a mixed‐effects model with subject random intercept and day as a categorical fixed effect demonstrated a significant overall time effect in the immediate‐treatment forearm (*p* = 0.002).

In the delayed‐start forearm, lesion coverage decreased from 2.95% ± 1.65% at baseline (Day 0) to 1.80% ± 1.79% at Day 45 (still pre‐treatment), which was not statistically significant (*p* = 0.3125). After treatment at Day 45, lesion burden declined by Day 90 (0.53% ± 0.30%, approximately 45 days post‐treatment; *p* = 0.0625) and remained significantly reduced at Day 365 (1.12% ± 0.85%, approximately 320 days post‐treatment; *p* = 0.0313). A mixed‐effects model similarly indicated a significant overall time effect in the delayed‐start forearm (*p* = 0.0039), reflecting the post‐treatment reduction following the Day 45 intervention.

Between‐arm comparisons at matched TST timepoints using paired tests showed no statistically significant differences at TST 0 (*p* = 0.18), TST 45 (*p* = 0.32), or the TST ~1‐year window (*p* = 0.90), indicating broadly similar trajectories across forearms once aligned by treatment timing.

### 3.3 Histological and Ultrastructural Analysis

**FIGURE 5:**
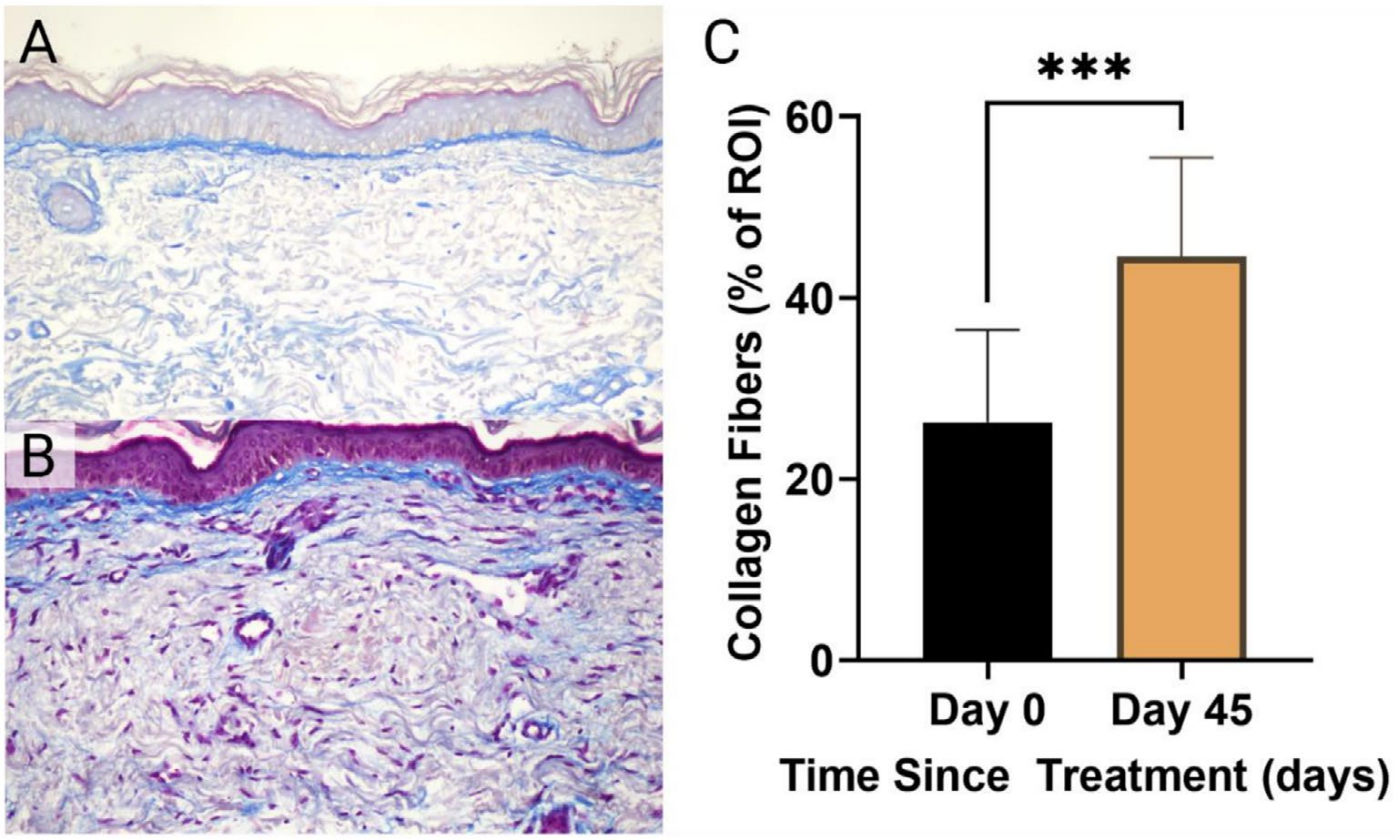
Increased collagen content in dermatoporotic skin following treatment with hyperdiluted CaHA–CMC. Representative Masson's Trichrome–stained histological sections at TST 0 (A) and TST 45 post‐treatment (B), demonstrating a visible increase in blue‐stained collagen fibers within the dermis. Quantitative analysis (C) confirmed a significant increase in collagen content from TST 0 to TST 45 (***p = 0.0009).

Pre‐treatment, the dermal ECM showed fragmented, disorganized collagen with reduced density and thickness, consistent with compromised structural integrity (Figure 5A). Post‐treatment, the ECM exhibited increased collagen density, thicker and more aligned fibers, and improved organization (Figure 5B). Quantitative histological analysis revealed a significant increase in collagen content following treatment. The mean percentage area of collagen, assessed with Masson's trichrome staining, increased from 26.26% ± 10.23 at baseline to 44.56% ± 10.93 at Day 45, representing a 69.7% increase between baseline and Day 45 (*p* = 0.0009) (Figure 5C).

**FIGURE 6:**
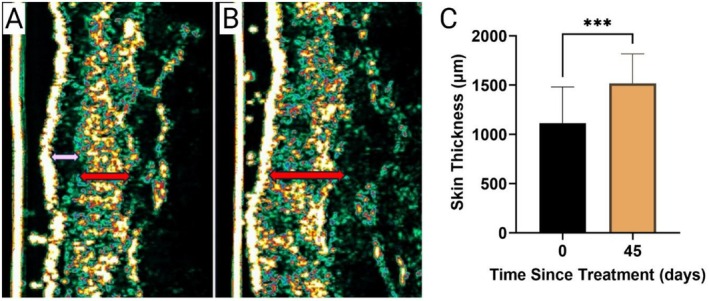
High‐frequency ultrasound analysis of dermal thickening following treatment with hyperdiluted CaHA–CMC. (A) Representative ultrasound image of the forearm at TST 0 showing reduced dermal thickness (red bar) and subepidermal hypoechogenic band (pink arrow) characteristic of dermatoporotic skin. (B) Corresponding image at TST 45 demonstrating visibly increased dermal thickness. (C) Quantitative comparison of skin thickness between TST 0 and TST 45, showing a significant increase in thickness following CaHA–CMC injection (***p = 0.0007).

Ultrasound measurements increased 36.05% from 1115.00 ± 366.00 μm at baseline to 1517.00 ± 300.40 μm 45‐days post treatment (*p* = 0.0007) (Figure 6). All patients experienced increases in skin thickness following treatment with CaHA‐CMC.

### 3.4 Qualitative Analysis

Treating investigators noted supportive qualitative changes in the dermatoporotic skin that included a reduction in visible blood vessels, a decrease in the number of lesions, and improvements in skin laxity and atrophy. These qualitative improvements were observed 45 days after treatment and persisted to some degree for approximately 1 year.

### 3.5 Adverse Events

Adverse events were mild, with one patient experiencing small nodules at the injection site, which resolved with massage within 30 days of onset. No other adverse events were reported.

## 4 Discussion

The present study provides histologic, ultrasonographic, and clinical evidence that hyperdiluted CaHA‐CMC promotes meaningful dermal regeneration in patients with forearm dermatoporosis. Treatment produced rapid and durable improvements in lesion burden, dermal thickness, and collagen density, consistent with the established biostimulatory profile of CaHA microspheres. These findings extend prior work demonstrating that CaHA‐CMC induces neocollagenesis through fibroblast mechanostimulation [27]. The current data suggest that these effects can be leveraged in dermatoporotic forearm skin.

Mechanistically, CaHA‐CMC acts through several complementary pathways. The CaHA microspheres provide a temporary scaffold that activates fibroblasts via integrin‐mediated mechanotransduction and YAP activation, resulting in collagen type I and III synthesis, elastogenesis, ECM remodeling, and angiogenesis [11, 13, 19, 27, 28]. Hyperdilution increases the distribution field and reduces direct volumization, allowing homogeneous stimulation across large, photo‐exposed surfaces such as the forearms [27, 29, 30]. The resulting ECM expansion and fiber reorganization align closely with the concept of stromal stabilization: reinforcement of the dermal matrix that improves shock absorption, dermal resilience, and microvascular support.

In dermatoporosis, a condition characterized by collagen fragmentation, reduced dermal thickness, and microvascular fragility, restoring ECM integrity has both structural and functional consequences. The observed 69.7% increase in collagen and 36.05% increase in dermal thickness suggests that CaHA‐CMC may re‐establish dermal tensile strength, thereby improving mechanical resistance to minor trauma and reducing purpura formation. The qualitative reduction in visible lesions may reflect improved perivascular support or decreased vascular permeability within the dermis. By reinforcing perivascular support and improving ECM structure, it is plausible that the propensity for minor trauma‐induced capillary rupture and subsequent purpura is reduced. Although angiogenesis was not directly assessed, it is possible that stromal stabilization mitigates ongoing microvascular injury, a hallmark of advanced dermatoporosis [1].

Clinically, these effects translated into a 73.4% mean reduction in visible lesion coverage sustained through approximately 1 year after a single treatment session, an outcome not previously reported in this population. These findings align with previous reports showing that CaHA‐CMC stimulates progressive ECM remodeling, a process that continues well beyond the initial treatment window, and whose clinical manifestations remain visible for 12 months or longer [21, 31]. The convergence of clinical and structural outcomes in this study suggests that dermal stabilization may play a central role in the long‐term persistence of benefit.

Although many CaHA‐based body treatment consensus recommendations describe multi‐session regimens (often three sessions) to optimize aesthetic outcomes such as laxity and texture, the present study was designed as an exploratory, proof‐of‐concept evaluation in a skin fragility syndrome (dermatoporosis) with objective endpoints (lesion burden, ultrasound dermal thickness, and histology) [32, 33]. A single treatment session was selected to minimize procedural burden and risk in an older fragile‐skin population, preserve feasibility for serial assessments including punch biopsies, and generate an interpretable initial efficacy signal to inform future regimen‐optimization studies. Accordingly, these results should not be interpreted as defining an optimal number of sessions; future studies should evaluate multi‐session protocols and retreatment intervals and stratify outcomes by baseline severity and secondary etiologies.

### 4.1 Limitations

This study has several limitations inherent to its exploratory, delayed‐start design. The small sample size restricts statistical power and generalizability, and observed effect magnitudes should be interpreted cautiously as they may overestimate population‐level responses. Because active systemic or topical corticosteroid therapy was an exclusion criterion, these findings may not generalize to patients with secondary dermatoporosis receiving ongoing corticosteroids. Active corticosteroid exposure is known to suppress dermal fibroplasia and collagen synthesis; therefore, response to biostimulatory interventions may differ in patients receiving ongoing corticosteroids [34, 35]. However, CaHA‐CMC–specific clinical data in active corticosteroid users are limited.

Although the delayed‐start contralateral design provided a short‐term internal control, the absence of a parallel placebo group limits the ability to fully isolate treatment effects from potential confounding influences such as behavioral modification or spontaneous improvement. Because the more severe forearm was assigned to immediate treatment (nonrandom allocation), early between‐arm comparisons may be influenced by baseline imbalance and regression‐to‐the‐mean. Additionally, the histological and ultrasound analyses taken 45 days post‐treatment likely do not capture the peak effect of biostimulation, which is known to persist for up to 1 year [21]. One participant missed the Day 45 evaluation, resulting in one incomplete bilateral pair and slightly reduced power for the early (0–45 day) comparative analysis. As with other photographic CAIA endpoints, outputs may remain sensitive to image acquisition conditions (lighting, exposure, white balance), skin tone, and evolution of purpura/bruising, and the segmentation parameters may be partially optimized to the photographic conditions used in this study. Finally, the follow‐up duration for delayed‐start arms (320 days post‐treatment) was slightly shorter than for immediate‐treatment arms (365 days), although both were analyzed within a common ~1‐year time window.

## 5 Conclusion

This prospective case series suggests that a single session of hyperdiluted CaHA–CMC can significantly thicken the dermis, increase collagen density, and reduce the clinical burden of lesions in forearm dermatoporosis for approximately 12 months. Quantitative histology, ultrasound, and digital image analysis converged to show durable aesthetic improvements in this patient subset. The procedure was well‐tolerated, with only minor, self‐limiting nodules reported in one participant and no serious adverse events. For patients with recurrent, cosmetically and functionally impactful purpuric lesions, these data suggest that single‐session hyperdiluted CaHA‐CMC may offer a durable, minimally invasive treatment option. While the uncontrolled design and small cohort limit generalizability, the magnitude and persistence of response are compelling. These findings justify randomized, blinded trials to refine dilution ratios, injection intervals, and long‐term safety. If validated in such studies, CaHA‐CMC could fill a critical therapeutic gap for patients with dermatoporosis.

## Author Contributions

**Ada Regina Trindade de Almeida:** conceptualization, methodology, validation, formal analysis, investigation, resources, writing – review and editing, supervision. **Maria Cortez de Sousa Marins Barbosa:** conceptualization, methodology, data curation, validation, formal analysis, investigation, resources, writing – original draft, writing – review and editing, supervision, visualization. **Alexandre Michalany:** conceptualization, methodology, validation, formal analysis, investigation, resources, writing – review and editing. **Alec D. McCarthy:** conceptualization, methodology, validation, data curation, formal analysis, investigation, resources, writing – review and editing, supervision, visualization.

## Ethics Statement

The authors confirm that the ethical policies of the Journal of Cosmetic Dermatology, as noted in the journal's author guidelines, have been fully adhered to. All procedures involving human participants were conducted in accordance with the Declaration of Helsinki. The study protocol was reviewed and approved by the Ethics Committee of Hospital do Servidor Público Municipal de São Paulo (Approval Date: 2022; Approval Number: CAAE 66468822.8.0000.5442). All patients provided written informed consent prior to publication, including consent for the use of clinical data, photographs, and biopsy samples for research and publication purposes.

## Conflicts of Interest

Dr. de Almeida is a speaker, researcher, and consultant for Abbvie and Merz Aesthetics. Dr. Barbosa and Dr. Michalany have no conflicts to report. Dr. McCarthy is employed by Merz Aesthetics.

## Supporting information


**Table S1:** Inclusion and Exclusion Criteria.

## Data Availability

The data that support the findings of this study are available on request from the corresponding author. The data are not publicly available due to privacy or ethical restrictions.
