# Supplementary material for: Calcium Hydroxylapatite‐Carboxymethylcellulose as a Treatment for Forearm Dermatoporosis: A Prospective, Delayed‐Start, Contralateral Case Series
Source: J Cosmet Dermatol. 2026 Apr 12;25(4):e70859. doi: 10.1111/jocd.70859 (PMC13071236; doi:10.1111/jocd.70859)
Supplement: Supplementary file 1 — Table S1: Inclusion and Exclusion Criteria. [file JOCD-25-e70859-s001.docx]

**Supporting Information**

**Calcium Hydroxylapatite-Carboxymethylcellulose as a Treatment for Forearm Dermatoporosis: A Prospective, Delayed-Start, Contralateral Case Series**

**Table S1. Inclusion and Exclusion Criteria**

| Inclusion Criteria | Exclusion Criteria |
| --- | --- |
| Patients with a clinical diagnosis of dermatoporosis in the forearm | Use of anticoagulants and antiplatelet agents (e.g., warfarin, heparin, aspirin, clopidogrel, dipyridamole, ticlopidine, cilostazol, prasugrel, ticagrelor, fondaparinux, bivalirudin, dabigatran, rivaroxaban, apixaban) |
| No previous specific treatment (topical vitamin C, topical retinoic acid, retinaldehyde, or alpha-hydroxy acids) for at least 30 days | Participants allergic to lidocaine, chlorhexidine, or calcium hydroxylapatite. |
| Signed informed consent | Active systemic or topical corticosteroids therapy |
